# Cyclic Hypoxia Induces Transcriptomic Changes in Mast Cells Leading to a Hyperresponsive Phenotype after FcεRI Cross-Linking

**DOI:** 10.3390/cells11142239

**Published:** 2022-07-19

**Authors:** Deisy Segura-Villalobos, Monica Lamas, Claudia González-Espinosa

**Affiliations:** Departamento de Farmacobiología, Centro de Investigación y de Estudios Avanzados (Cinvestav), Unidad Sede Sur. Calzada de los Tenorios No. 235, Col. Granjas Coapa, Tlalpan, Mexico City 14330, Mexico; deisy.segura@cinvestav.mx (D.S.-V.); mlamas@cinvestav.mx (M.L.)

**Keywords:** mast cells, cyclic hypoxia, hyperresponsive phenotype, degranulation, cancer, tumor microenvironment, FcεRI receptor

## Abstract

Mast cells (MCs) play important roles in tumor development, executing pro- or antitumoral functions depending on tumor type and tumor microenvironment (TME) conditions. Cyclic hypoxia (cyH) is a common feature of TME since tumor blood vessels fail to provide a continuous supply of oxygen to the tumor mass. Here, we hypothesized that the localization of MCs in cyH regions within solid tumors could modify their transcriptional profile and activation parameters. Using confocal microscopy, we found an important number of MCs in cyH zones of murine melanoma B16-F1 tumors. Applying microarray analysis to examine the transcriptome of murine bone-marrow-derived MCs (BMMCs) exposed to interleaved cycles of hypoxia and re-oxygenation, we identified altered expression of 2512 genes. Functional enrichment analysis revealed that the transcriptional signature of MCs exposed to cyH is associated with oxidative phosphorylation and the FcεRI signaling pathway. Interestingly, FcεRI-dependent degranulation, calcium mobilization, and PLC-γ activity, as well as *Tnf-α*, *Il-4*, and *Il-2* gene expression after IgE/antigen challenge were increased in BMMCs exposed to cyH compared with those maintained in normoxia. Taken together, our findings indicate that cyH causes an important phenotypic change in MCs that should be considered in the design of inflammation-targeted therapies to control tumor growth.

## 1. Introduction

Mast cells (MCs) are tissue-resident secretory cells with versatile functions in innate and adaptive immune responses [1]. Morphologically, MCs are characterized by their abundant cytoplasm filled with a number of electrodense granules that store preformed active molecules which are differentially released upon proper activation [2]. The best-characterized pathway for MC activation depends on the crosslinking of the high-affinity immunoglobulin E (IgE) receptor (FcεRI). In this cell type, the receptor is composed of one α-chain that binds the Fc fragment of IgE, a β-chain, and two γ-chains, which are important for signal transduction [3]. FcεRI-dependent activation of MCs initiates when a specific antigen (Ag) brings together FcεRI-bound IgEs, triggering receptor clustering that initiates a complex intracellular signaling cascade which involves phosphorylation of the immunoreceptor tyrosine-based activation motifs (ITAMs) found on the β- and γ- chains by Src-family tyrosine kinases, such as Lyn and Fyn. Subsequently, Syk kinase is translocated to phosphorylated ITAMS and, in turn, phosphorylates adapters and other molecules responsible for signal amplification [4,5,6]. FcεRI triggering leads, among other events, to the activation of PLCγ and increased intracellular calcium (Ca^2+^) mobilization that culminates in the release of active mediators through a process known as degranulation [7,8]. Among the mediators released by MCs in response to the activation of FcεRI are preformed molecules, such as histamine and the enzyme β-hexosaminidase. De novo synthesized mediators, such as prostaglandins, cytokines, and growth factors are also released [9,10]. These active compounds modulate host-protective immune responses against potentially harmful host agents and conditions [1,11]. Moreover, MCs have been implicated in the pathophysiology of several diseases, including allergies and chronic inflammation-associated diseases such as cancer, in which MCs have been considered reactive bystanders and amplifiers of the inflammatory reaction [12].

Evidence indicates a close relationship between MCs and tumors in both human and animal cancer models [13,14]. Indeed, there is overwhelming evidence that MCs can infiltrate different types of solid tumors, including melanoma [13,15,16]. Once there, they are known as tumor-associated MCs (TAMCs) and might play a critical role in promoting and/or limiting tumorigenesis [17,18]. It has been proposed that, as occurs with other innate immune cells, such as macrophages that can polarize towards M1-like (protumor) or M2-like (antitumor) cells [19,20], TAMCs could also present particular phenotypes. It is known that the dual role of MCs in tumor development depends on tumor type and stage, as well as the stimuli these cells receive from the tumor microenvironment (TME), but the mechanisms by which MCs can polarize toward one of the two phenotypes (or a different one) have not been fully understood.

Given that MCs possess high phenotypic plasticity, it has been proposed that TAMCs could differentiate into at least two phenotypes within the tumor: MC1 (antitumoral) and MC2 (protumoral), and this phenotypic change could occur in response to stimuli from the TME [13,21,22]. TME is characterized by hypoxia, an intrinsic feature of all solid tumors [23]. The hypoxic TME derives from the imbalance of oxygen (O_2_) consumption versus supply due to the high proliferation rate of tumor cells and chaotic tumor angiogenesis [24,25]. This dysfunctional vasculature leads to cyclic hypoxia (cyH), a state characterized by periods of hypoxia/re-oxygenation because of temporal fluctuations of O_2_ levels within the TME [26]. It has been documented that MCs respond to hypoxia by changing their cytokine secretory profile and increasing the expression of hypoxia-associated markers, such as hypoxia-inducible factor (HIF)-1α, the chemokine CC-motif ligand 2 (CCL-2), and the vascular endothelial growth factor (VEGF), allowing them to adapt and survive to this metabolic stress [27,28,29,30].

The present study aimed to assess the hypothesis that MCs are subjected to cyH within solid tumors and that this imbalance of O_2_ levels can trigger phenotypic plasticity in MCs. Utilizing the B16-F1 murine melanoma model and bone-marrow-derived mast cells (BMMCs), we present evidence that TAMCs are present in regions subjected to cyH inside melanoma tumors. In addition, cyH modifies the transcriptome of BMMCs, altering the expression profile of 2512 genes. Interestingly, upregulated genes were associated with oxidative phosphorylation and the FcεRI signaling pathway. Finally, we found that FcεRI-dependent degranulation and Ca^2+^ mobilization were potentiated in BMMCs exposed to cyH. Overall, these findings confirm that cyH induces major phenotypic plasticity in MCs, leading to a hyperresponsive phenotype upon antigenic challenge. This study reinforces the notion that certain microenvironment conditions, such as cyH, reshape the phenotype of immune cells present in the tumor and suggests that thresholds to activate specific pathways (such as the triggered by IgE/Ag complexes) are altered in TAMCs.

## 2. Materials and Methods

### 2.1. Animals

Eight- to ten-week-old C57BL/6J mice (stock no. 000664) from The Jackson Laboratory were used in the current study. Animals were housed under controlled temperature (22–24 °C) and humidity conditions in a 12 h light/12 h dark cycle with *ad libitum* access to food and water. All experimental procedures were performed according to our Institutional Committee for the Care and Use of Laboratory Animal (CICUAL, protocols numbers 237-15 and 15-12) and following the National Institutes of Health (NIH) guidelines for the use and care of laboratory animals.

### 2.2. Reagents and Antibodies

Antitryptase antibody (cat. Number: 32889) was purchased from Santa Cruz Biotechnology (Dallas, TX, USA). Antibodies against pimonidazole and CD31 (Cat. Number 550274) were from Hypoxyprobe, Inc. (Burlington, MA, USA) and BD Biosciences (Franklin Lakes, NJ, USA), respectively; secondary antibodies donkey antirabbit Alexa 647 (cat. Number: A31573) and donkey antirat Alexa 594 (cat. Number: A21209) were acquired from Molecular Probes (Eugene, OR, USA). Tissue-Tek O.C.T. was from Sakura Finetek (Torrance, CA, USA). β-mercaptoethanol (β-ME), Tri Reagent, agarose, 2′7′-dichlorodihydrofluorescein diacetate (DCFH_2_-DA), IGEPAL CA-630, antidinitrophenyl (anti-DNP) IgE (clone SPE-7), DNP coupled to human serum albumin (DNP-HSA), Ca^2+^ ionophore A23187, protein kinase C (PKC) activator phorbol 12-myristate 13-acetate (PMA), Fura 2-AM, phospholipase C (PLC) inhibitor U73122, phosphate-buffered saline (PBS), and all salts for buffers and solutions were obtained from Sigma-Aldrich (St. Louis, MO, USA). Cell culture media (DMEM and RPMI 1640) and other reagents such as fetal bovine serum (FBS), antibiotic/antimycotic mixture, pyruvate, nonessential amino acids, bovine serum albumin (BSA), and EDTA-trypsin were from Invitrogen (Waltham, MA, USA), except for recombinant murine interleukin 3 (IL-3) that was from PeproTech (Rocky Hill, NJ, USA).

### 2.3. Culture of B16-F1 Melanoma Cells and Tumor Generation

Murine B16-F1 melanoma cell line was purchased from ATCC (CRL-6323) and cultured according to the instructions provided. Briefly, B16-F1 cells were cultured in DMEM medium supplemented with 10% heat-inactivated FBS, penicillin 100 U/mL, and streptomycin 100 μg/mL. Cell culture medium was changed twice weekly, and cells were maintained at 37 °C and 5% CO_2_ until reaching 80% confluence. Then, adherent cells were detached by adding a 0.05% EDTA-trypsin solution. Digestion was stopped with fresh culture medium supplemented with FBS, and cells were retrieved by centrifugation. Cells were passaged at least two times before use.

For tumor generation, C57BL/6J mice were subcutaneously inoculated into the left ear pinna with $0.5\times 10^{6}$ melanoma cells as previously described [27,28]. Animals were kept in controlled conditions and tumor development was monitored until a black tumor mass was visible in the pinna of the mouse (around 20 days). Then, to detect hypoxic zones, mice were administered intraperitoneally (i.p.) with 60 mg/kg of pimonidazole or saline. After 30 min, animals were euthanized by CO_2_ inhalation, and tumors were quickly removed. Then, they were placed in a 4% PFA solution for 48 h, and later in a 30% sucrose solution for 48 h, at 4 °C. Finally, tumors were embedded in freezing medium Tissue-Tek and sectioned into 30-μm-thick sections using a cryostat (Model Hyrax C25) from Carl Zeiss (Jena, Germany).

### 2.4. Immunofluorescence and Confocal Microscopy in Tumor Biopsies

Tumor slices (30 μm width) were placed in P24 cell culture and incubated with a solution of NH_4_Cl (50 mM) for 15 min to decrease autofluorescence. After this time, slices were washed two times with a solution of PBS 1X-0.1% Triton X-100 (PBS-T) to remove the excess of NH_4_Cl. Then, the tumor samples were permeabilized and blocked with a solution of 3% BSA, 5% donkey serum, and 0.3% Triton X-100 for 2 h at room temperature. For detection of hypoxic areas, tumor slices were incubated overnight at 4 °C with a primary FITC-conjugated antibody solution (1:150). In the same preparations, rabbit antitryptase Ab (1:50) and rat anti-CD31 Ab (1:100) were used to detect MCs and blood vessels, respectively. After that, slices were incubated in a 1:500 dilution of Alexa-647 donkey antirabbit and Alexa-546 donkey antirat secondary Abs for 2 h at room temperature. Nuclei were stained with DAPI (1:500) for 5 min. Finally, excess DAPI was removed by washing with PBS-T three times, and the samples were mounted with DABCO on microscope slides. Images were acquired using a Carl Zeiss Airscan LSM-800 confocal microscope with a 10X objective, and a 40X objective was used for magnification. In all assays, negative controls were performed in parallel to each experiment, omitting the incubation with primary Abs [31]. In all cases, no significant signal was observed in the presence of only the secondary Abs (Figure S1), indicating a high specificity of the antibodies used. Images obtained were analyzed using the Zen 2.3 SP1 software, Blue Edition, and ImageJ (NIH) software program. Colocalization analysis was performed using the Manders Coefficients (M1 and M2), which calculate the percentage of total signal from one channel (channel 1) that overlaps with the signal from the other (channel 2) and vice versa [32].

### 2.5. Generation of BMMCs Cultures

Bone marrow cells were isolated from the tibia of 4- to 6-week-old mice. BMMCs were differentiated in vitro in RPMI medium supplemented with 20 ng/mL IL-3, 10% heat-inactivated FBS, 25 mM HEPES, 50 μM β-ME, 0.1 mM nonessential amino acids, 1mM pyruvate, 100 U/mL penicillin, and 100 μg/mL streptomycin. The culture medium was changed every 7 days removing adherent cells each time, and cell cultures were maintained at a density of $1.0-1.5\times 10^{6}$ cells/mL in a humidified 37 °C incubator with 5% CO_2_ for at least 5 weeks.

To ensure the differentiation and purity of BMMCs, the expression of FcεRI on cell cultures was evaluated by flow cytometry (Beckman Coulter, Brea, CA, USA) using a specific antibody against the α subunit of FcεRI (Cat. number: 17-5898-80). Only cultures with at least 98% of cells positive for this receptor were used. Additionally, the functionality of BMMCs was assessed by determining β-hexosaminidase release and cytokine mRNA expression in response to the Ca^2+^ ionophore A23187 and the PKC activator PMA. All the experiments were performed with cultures between 6 and 8 weeks of age at a final density of $2.0\times 10^{6}$ cells/mL.

### 2.6. In Vitro Protocol for Cyclic Hypoxia

Hypoxic conditions were induced in vitro using a Memmert Model INC108 (Büchenbach, Germany) incubator, which was gassed with 95% N_2_ and 5% CO_2_ to produce an O_2_ concentration of 1%. For the generation of cyH conditions, mature BMMCs were seeded in P24 cell culture plates and exposed to four cycles consisting of 1 h hypoxia (1% O_2_) alternated by 30 min re-oxygenation (21% O_2_), as previously reported in studies with endothelial cells [33]. To standardize cyH conditions, cells were harvested at the end of each period of hypoxia and re-oxygenation (H1, R1, H2, R2, H3, R3, H4, R4), H1 cells being those that were exposed to 1 h of hypoxia, R1 cells those subjected to 1 h of hypoxia plus 30 min of re-oxygenation, and so on until the end of the cyH protocol (6 h total). On the other hand, normoxic cells (N) were exposed to the same conditions but in normal O_2_ concentrations (21%) for 6 h. When needed as a control, BMMCs were incubated for an uninterrupted period of 2.5 h under 1% O_2_ to induce chronic hypoxic (chH) conditions.

### 2.7. Viability Test

The viability of BMMCs subjected to the cyH protocol was assessed using the Muse™ Count & Viability Kit (Millipore) according to the manufacturer’s instructions. Briefly, one million normoxic or hypoxic BMMCs were stained by mixing them with Muse™ Count & Viability reagents at a 1:10 dilution. Cells were incubated for 5 min at room temperature and then loaded onto the Muse™ Cell Analyzer to generate the corresponding dot plot with the percentage of live and dead cells for each sample.

### 2.8. RNA Extraction and RT-PCR

Total RNA was extracted from cells using TRIzol according to the provider’s instructions. Isolated RNA was re-suspended in 7 μL of RNA secure solution (Thermo Fisher Scientific, Waltham, MA, USA) and the quality and quantity of total RNA were evaluated by spectrophotometric analysis using a NanoDrop 2000 from Thermo Scientific. cDNA was synthesized with 2 μg of RNA using the Revert Aid First Strand cDNA Synthesis Kit (Thermo Fisher Scientific) following the provided instructions, and samples were stored at −70 °C until used. Expression of *Hif-1α*, *Vegf*, *Tnf-α*, *Ccl-2*, *Il-6*, *Il-4*, *Il-2*, *Tgf-β*, and *Gapdh* genes was determined by semiquantitative PCR using 5 μL of each cDNA sample and specific oligonucleotides, whose sequences are listed in Table S1. Annealing temperatures were used as described in the pertinent references (see Table S1) and following the optimal cycle parameters previously reported for amplification in BMMCs [34]. Finally, PCR products were resolved by 2% agarose gel electrophoresis and visualized with ethidium bromide. For quantification, the pixel intensity of each band was determined using the MiniBIS Pro from DNR Bio-Imaging systems (Neve Yamin, Israel) and normalized to *Gapdh* used as the housekeeping gene.

### 2.9. Determination of ROS Production

Intracellular production of reactive oxygen species (ROS) was determined by the method previously reported in [35] with some modifications. Briefly, after normoxia or cyH protocol (H1 to R4), one million cells per condition were collected in a 1.5-mL flat-top Eppendorf tube and centrifuged at 240× *g* for 4 min at room temperature to remove the cell culture medium. BMMCs were then re-suspended in 500 μL of Tyrode’s buffer and incubated with DCFH_2_-DA (10 μM final concentration) for 30 min at 37 °C and 21% O_2_. Following incubation, cells were recovered by centrifugation (12,000× *g* at 4 °C, 5 min), and the supernatant was discarded by aspiration with a vacuum system. Then, the cell pellet was disrupted by adding 300 μL of IGEPAL (0.1%) at 4 °C and pipetting vigorously. Subsequently, tubes were centrifuged at 12,000× *g* at 4 °C for 5 min, and 200 μL of the supernatant was placed in a 96-well black plate. Finally, the fluorescence of each sample was measured on a BioTek Microplate luminometer, model FLx800 from BioTek (Winooski, VT, USA) with λ_excitation/emission_ = 488/565 nm. Basal ROS generation was measured from BMMCs exposed to normoxic conditions. Intracellular ROS generation is shown as arbitrary units of fluorescence with respect to basal. In some specific experiments, BMMCs were incubated in chH for 2 h followed by 1 h of re-oxygenation, and cells incubated under normoxic conditions for 3 h were used as control. After that, ROS levels were determined as described above.

### 2.10. Microarray Assay

#### 2.10.1. Total RNA Isolation

Total RNA was isolated as described in Section 2.8. RNA samples were obtained from three different cell cultures, which were exposed to normoxia (21%O_2_ for 2.5 h) or cyH (H2). Then, the purity and integrity of total pooled RNA were analyzed both by agarose gel electrophoresis and by spectrophotometric analysis, and only samples with a 260/280 ratio close to 2.0 were used. Microarray assay was performed at the Microarray Unit of Cellular Physiology Institute (UNAM, Mexico City, Mexico), using the Mouse 65-mer oligo library from Sigma-Genosys oligo sets, which contains 22,000 gene-specific oligonucleotide probes representing 70% of the mouse genome. All data obtained in this study have been deposited in the NCBI’s Gene Expression Omnibus (GEO) and are available on the GEO website with the accession number GSE201828.

#### 2.10.2. Probe Preparation and Hybridization to Arrays

These processes were performed as described in previous works [36,37], with some modifications. Briefly, 10 μg of total pooled RNA were used for cDNA synthesis, incorporating dUTP-Alexa555 or dUTP-Alexa647. This was performed employing the First-Strand cDNA labeling kit from Invitrogen. The incorporation of the fluorophore was analyzed using the absorbance at 555 and 650 nm for Alexa555 and Alexa647, respectively. Then, equal quantities of label cDNA were hybridized using hybridization solution UniHyb (TeleChem International INC) to the 22 thousand oligos mouse arrays for 14 h at 42 °C. Each sample was labeled as follows: Alexa555 labeling was used for normoxic cells while Alexa647 labeling was used for hypoxic cells.

#### 2.10.3. Data Acquisition and Analysis of Array Images

Acquisition and quantification of array images were performed in a GenePix 4100-A reader with its accompanying software GenePix from Molecular Devices. For each spot, the Alexa555 and Alexa647 density mean value, background mean value, and signal cross channel lees normalization value (by subgrids) were calculated with the software ArrayPro Analyzer from Media Cybernetics.

#### 2.10.4. Microarray Data Analysis

This was performed with the free software genArise, developed in the Computing Unit of Cellular Physiology Institute of UNAM (http://www.ifc.unam.mx/genarise/ (accessed on 6 December 2019)). With this software, differentially expressed genes (DEGs) between normoxia and cyH samples were selected by calculating the intensity-dependent Z-score according to the following equation, as previously reported [37]:

(1) $$Zi=\left(\frac{Ri-mean\left(R\right)}{SD\left(R\right)}\right)$$

where *Zi* is the Z-score for each element, *Ri* is the log-ratio for each element, and *SD(R)* is the standard deviation of the log-ratio. Based on this criterion, the elements with a Z-score > 1.5 were considered DEGs [36]. In addition, log2 (fold change) and -log10 (adjusted P) were calculated for each gene, and a volcano plot was drawn using these parameters. The threshold was set at *p*-value ≤ 0.05 (expressed as the logarithm) which was marked by a gray dotted line in the volcano plot. Dots located under the threshold symbolize genes that did not change or that are false positives.

#### 2.10.5. Functional Enrichment Analysis

To further understand the potential biological roles of upregulated DEGs related to cyH, we performed a functional enrichment analysis using the open-access Database for Annotation, Visualization, and Integrated Discovery (DAVID) [38,39]. Gene Ontology (GO) terms such as cellular components, biological process, and molecular functions were analyzed. In addition, enrichment pathways using the KEGG (Kyoto Encyclopedia of Genes and Genomes) database collections were also included. For all these analyses, Fisher’s exact test was used and *p*-values < 0.05 were considered statistically significant.

### 2.11. Quantitative Real-Time PCR

To validate microarray results, we employed SYBR green-based quantitative PCR (qPCR) that was performed on a thermocycler PikoReal 96 (Thermo Scientific), using Maxima SYBR Green/ROX qPCR Master Mix (2x) from Thermo Fisher (Waltham, MA, USA). The amplification of selected genes was performed with 150 ng of cDNA and 0.5 μM of the following specific mouse primers: *Map2k6*, 5′-ATGTCTCAGTCGAAAGGCAAG-3′ (forward) and 5′-TTGGAGTCTAAATCCCGAGGC-3′ (reverse); *Pla2g4a*, 5′-CAGCACATTATAGTGGAACACCA-3′ (forward) and 5′-AGTGTCCAGCATATCGCCAAA-3′ (reverse); *Fcer1g*, 5′-CTCCTTTTGGTGGAACAAGC-3′ (forward) and 5′-GGGTAAGGACAATACCATACAAAAA-3′ (reverse); and *Gapdh*, 5′-ATTGTGGAAGGGCTCATGAC-3′ (forward) and 5′-AGTGGATGCAGGGATGATGT-3 (reverse). All these sequences were selected and taken from PrimerBank (https://pga.mgh.harvard.edu/primerbank/ (accessed on 17 November 2021)), except for *Fcer1g*, whose sequence has been previously reported [40]. Thermal cycler conditions were 95 °C for 10 min and 40 cycles of 15 s at 95 °C followed by 40 s at 60 °C and an elongation phase at 72 °C for 20 s. All experiments were performed in triplicate and a reaction mixture without cDNA was used as a negative control in each run. In addition, ROX was used as a passive reference dye to normalize the fluorescence intensity of SYBR green dye. Obtained mRNA levels were normalized to *Gapdh* expression, and the relative expression of each gene was calculated using the 2^−∆∆Ct^ method [41].

### 2.12. Degranulation Assay

Degranulation was assessed by measuring β-hexosaminidase release as described in [42] with some modifications. Briefly, BMMCs were incubated in normoxia (2.5 h at 21% O_2_), cyH (H2), or chH (2.5 h at 1%O_2_) and sensitized with 100 ng/mL IgE anti-DNP one hour before the end of each protocol. After that, cells were centrifuged and re-suspended in Tyrode’s/BSA buffer ($2.0\times 10^{6}$ cells/mL). Different concentrations of the specific Ag DNP-HSA (1, 3, 9, and 27 ng/mL) or vehicle (basal release of β-hexosaminidase) were added to the cells, which were subsequently incubated at 37 °C for 30 min in normoxic conditions. In another set of experiments, BMMCs exposed to N or cyH were pretreated with vehicle (DMSO, final concentration 0.1%) or different concentrations of U73122 (0.0001, 0.001, 0.01, 0.1 or 1 μM) for 15 min and then stimulated with Ag (9 ng/mL). In addition, a different stimulus than Ag was used. In this specific case, normoxic or hypoxic cells were incubated with 1 μM of both PMA and A23187 for 30 min.

After all these treatments, cells were centrifuged for 10 min at 4 °C and 60 μL of each cell supernatant was transferred into a 96-well plate followed by 40 μL of the substrate p-nitrophenyl-N-acetyl-β-D-glucosamide (P-NAG). The reaction between β-hexosaminidase and P-NAG was performed at 37 °C for 1 h and finished by adding 120 μL of the stop buffer (Na_2_CO_3_ 0.1 M/Na_2_HCO_3_ 0.1 M). The absorbance of each sample was measured by spectrophotometry at 405 nm using a microplate reader Tecan Sunrise (Männedorf, Switzerland). The release of β-hexosaminidase (expressed as % of total activity) was obtained by dividing the supernatant absorbance of each sample and the absorbance of the unstimulated cell pellet, which was solubilized with 0.05% Triton X-100.

### 2.13. Determination of Intracellular Calcium

Intracellular Ca^2+^ concentration [Ca^2+^]*i* was determined in $10\times 10^{6}$ normoxic or hypoxic IgE-sensitized BMMCs (1 h). Cells were labeled with Fura 2-AM (5 μM) for 30 min at 37 °C and 21% O_2_ and, after that, were washed two times and re-suspended in 2 mL of Tyrode´s/BSA buffer. Next, Fura 2-AM-loaded BMMCs were placed in the cuvette, and the changes in fluorescence were determined in intervals of 1.16 s with a λ_excitation/emission_ = 340/510 nm using a spectrofluorometer Fluoromax 3 (Jobin Yvon, Horiba). Throughout the experiment, the cells were held at 37 °C and under agitation. Basal fluorescence was recorded for 100 s followed by fluorescence after the addition of Ag (27 ng/mL) which was recorded for 500 s. At the end of the experiments, the maximum fluorescence (*F_max_*) was recorded by measuring fluorescence after the addition of Triton (10%), while the minimum fluorescence (*F_min_*) was obtained by adding the Ca^2+^ chelating agent EGTA (200 mM). Finally, [Ca^2+^]*i* was calculated using the method of Grynkiewicz [43] as follows:

(2) $$\left[{\mathrm{Ca}}^{2+}\right]i=Kd\left(\frac{F-F_{min}}{F_{max}-F}\right)$$

where *Kd* is the effective dissociation constant of Fura 2-AM (224 nM).

The data was normalized and reported as the fold-change of [Ca^2+^]*i* with regard to basal fluorescence. In addition, the data were fitted to a monoexponential function to calculate the rate constant (K).

### 2.14. Statistical Analysis

Results are numerically expressed as mean ± SEM of at least three independent experiments. All statistical comparisons were performed between normoxia and cyH unless otherwise specified. Cell cultures were assigned randomly to experimental groups by simple random sampling. The normality distribution of data was validated with the Shapiro–Wilk test. The comparability among experimental conditions was assessed by parametric and nonparametric tests, as appropriate. When F achieved minimal statistical significance, the Tukey post hoc test was used for multiple comparisons. For all the experiments, data were considered significant if *p* < 0.05. All fits were performed using GraphPad Prism 8 (GraphPad Software, San Diego, CA, USA).

## 3. Results

### 3.1. MCs Can Be Found in Cyclic Hypoxia Regions within Murine B16 Melanoma Tumors

**Figure 1:**
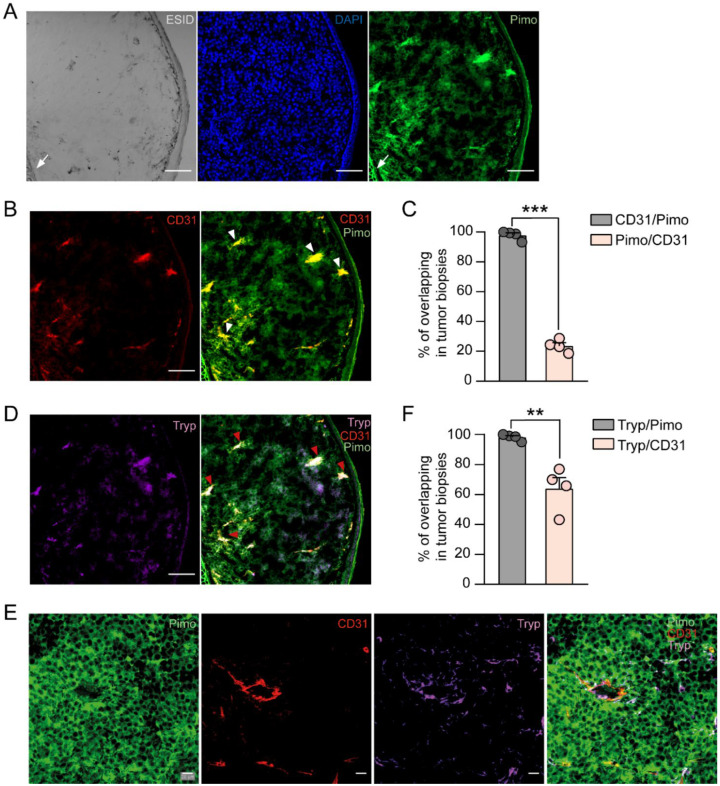
MCs are located in zones subjected to cyclic hypoxia within murine B16 melanoma tumors. Three to four weeks after inoculation of B16-F1 cells on mouse ear pinna, tumors were excised and processed as described in the Materials and Methods section. Thirty μm-thick slices were stained with specific antibodies against pimonidazole (Pimo), the platelet endothelial cell adhesion molecule (CD31), and tryptase (Tryp). (**A**) Representative ESID confocal image showing typical pinna morphology and the tumor mass (the ear cartilage is indicated by a white arrow). Cell nuclei and hypoxic regions were detected using DAPI (blue) and antibodies against pimonidazole (Pimo, green), respectively. (**B**) Tumor vasculature stained with an anti-CD31 antibody (red) and hypoxic areas with Pimo (green), showing colocalization between both fluorescent dyes (white arrowheads). Scale bar, 100 μm; objective 10×. (**C**) Quantification of the overlap between CD31/Pimo and Pimo/CD31 fluorescent signals. (**D**) Representative confocal images of tumor-infiltrating MCs stained with an antibody against tryptase (Tryp, purple) and their colocalization with CD31 and Pimo-positive regions (red arrowheads). Scale bar, 100 μm; objective 10×. (**E**) Magnified confocal images showing a large blood vessel (CD31+, red) in the Pimo-positive regions that are surrounded by Tryp-positive MCs. Scale bar, 20 μm; objective 40×. (**F**) Quantification of the overlap between Tryp/Pimo and Tryp/CD31 signals. C and F panels, unpaired t-test, *** *p* < 0.001 and ** *p* < 0.01, *n* = 4 tumor biopsies from four independent animals.

It has been shown that MCs infiltrate murine melanoma tumors and are mainly located in hypoxic zones within B16-F1 murine melanoma tumor tissue [28]. However, whether these cells are exposed to cyH has not been reported. Evidence obtained with distinct real-time methodologies applied to the analysis of tumors in vivo (reviewed in [44]) indicates that O_2_ levels fluctuate within solid tumors, mainly due to abnormal angiogenesis characterized by disorganized and tortuous vessels that tend to have blood leaks, leading to patterns of hypoxia/re-oxygenation inside the tumor [26]. Those studies have allowed the implementation of some anatomical criteria to define that a certain tissue zone is potentially subjected to cyH [26,45]. With this in mind, we assessed the possibility that melanoma tumors may present zones suffering cyH, and MCs could be subjected to those conditions, too. Using confocal microscopy and the hypoxia marker pimonidazole on tumors removed from the ear pinna, we were able to detect normoxic (pimonidazole negative) and hypoxic (pimonidazole positive) zones (Figure 1A). As expected, it was observed that ear cartilage, a nonvascular connective tissue, was strongly stained with pimonidazole (Figure 1A, white arrows).

Next, we analyzed the tumor vasculature in melanoma biopsies using the blood vessel marker CD31. Microscopic analysis revealed that blood vessels colocalize with hypoxic areas within the melanoma tumor (Figure 1B, white arrowheads), suggesting the existence of conditions associated with cyH [44]. Manders coefficients were calculated to determine the colocalization of CD31 (blood vessels) and pimonidazole (hypoxic zones). The percentage overlap between CD31 and pimonidazole signals was 97.8 ± 1.6%, suggesting that almost all detected blood vessels in tumor biopsies were located in hypoxic areas, whereas that overlapping of pimonidazole and CD31 was significantly lower (pimonidazole/CD31 = 23.6 ± 2.1%), indicating that large hypoxic tumor areas were not irrigated (Figure 1C). Taken together, these results confirm the existence of a poorly functional vascular network and suggest a different vascular distribution in normoxic and hypoxic areas in this tumor.

To analyze the presence of MCs in zones potentially subjected to the cyH of melanoma tumors associated with this abnormal vasculature, we used an antitryptase antibody to detect this cell type. Tryptase-positive cells were detected in CD31 and pimonidazole-positive areas (Figure 1D, red arrowheads). A magnified image shows that tryptase-positive MCs (purple) surround a large blood vessel (red) in a hypoxic area (green) (Figure 1E). Overlap between tryptase and pimonidazole signals was close to 100%, corroborating that MCs are located in hypoxic areas within the melanoma tumor [28]. Interestingly, the overlapping between tryptase and CD31 signals was significantly lower (tryptase/pimonidazole = 97.8 ± 1.6% versus tryptase/CD31 = 63.9 ± 7.3%; Figure 1F), indicating that not all MCs are close to blood vessels. Obtained results provide experimental evidence that MCs are localized in tumor zones subjected to cyH, indicating that tumor-associated MCs (TAMCs) are exposed to and probably activated by cyH within the melanoma tumor niche.

### 3.2. Cyclic Hypoxia Increases mRNA Levels of Hypoxia-Associated Genes and ROS Production in BMMCs

**Figure 2:**
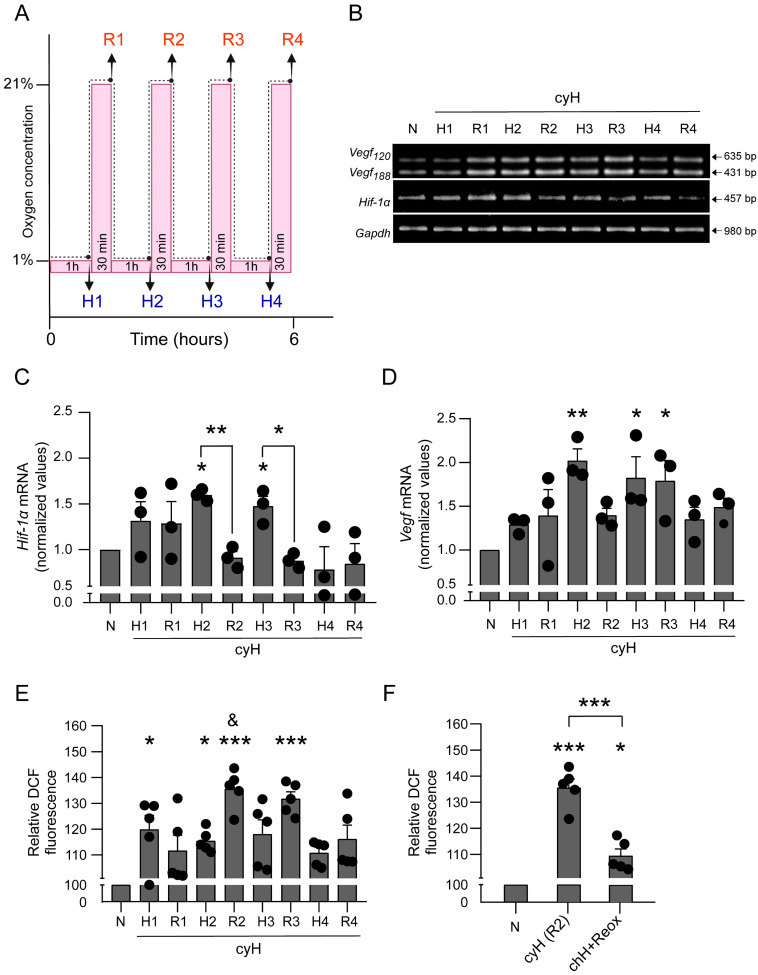
Cyclic hypoxia increases *Hif-1α* and *Vegf* mRNA levels and promotes ROS production in early re-oxygenation periods in BMMCs. (**A**) Experimental protocol of cyH indicating the time and the hypoxia/re-oxygenation cycles to which BMMCs were exposed. Arrows indicate the exact point where cells were harvested. The total time of the protocol (hypoxic + re-oxygenation periods) was accomplished in 6 h. (**B**) Two million BMMCs were incubated in normoxia (6 h at 21%O_2_, N) or cyH according to the protocol described in A. Total mRNA was purified at indicated points for RT-PCR analysis of *Hif-1α* and *Vegf* expression. A representative image from three distinct experiments performed with independent cultures of BMMCs is shown. (**C**) Densitometric quantification of *Hif-1α* and (**D**) *Vegf* mRNA normalized to *Gapdh* expression. (**E**) One million BMMCs were treated as in A and then loaded with DCF-DA for 30 min. The fluorescence intensity was taken as a measurement of ROS production and normalized with the levels of ROS in N. (**F**) One million BMMCs were exposed to N (3 h at 21% O_2_), cyH (two cycles of 1 h at 1% O_2_ followed by 30 min at 21% O_2_), or chH+Reox (2 h at 1% O_2_ followed by 1 h at 21% O_2_). Then, ROS production was evaluated as in E. One-way ANOVA, * *p* < 0.05, ** *p* < 0.01, *** *p* < 0.001 versus N and specified groups; and ^&^ *p* < 0.05 versus H2, *n* = 3–5 experiments for each condition using different BMMCs cultures.

Several studies have evidenced different consequences of cyH in endothelial and cancer cells [33,46,47,48]; however, the effects of that condition in MCs have not yet been explored. Thus, to analyze the molecular changes triggered in MCs by cyH, we used BMMCs to standardize an in vitro protocol for this condition, consisting of four cycles of 1 h at 1% O_2_ followed by 30 min re-oxygenation (Figure 2A). To validate this protocol, we evaluated several effects that have been described in other cells [33,46,47,48], such as the relative expression of the hypoxia markers *Hif-1α* and *Vegf* mRNAs at each period of hypoxia (H1, H2, H3, H4) and re-oxygenation (R1, R2, R3, R4). As shown in Figure 2B, the expression level of both genes was significantly higher after two and three periods of hypoxia (H2 and H3) compared with those observed in normoxic conditions, and no differences were observed in the fourth cycle of hypoxia/re-oxygenation. Interestingly, *Hif-1α* expression was diminished in the second and third periods of re-oxygenation (R2 and R3), compared with its previous hypoxia periods (one way-ANOVA; F_(8, 18)_ = 3.189; *p* = 0.019; Figure 2C). On the other hand, *Vegf*, a target gene of the HIF-1α complex, maintained its increased expression until R3, returning to basal levels in the fourth cycle of hypoxia/re-oxygenation (one way-ANOVA; F_(8, 18)_ = 3.464; *p* < 0.014; Figure 2D). These transcriptional changes did not induce significant cell death, since viability was greater than 95% in cells exposed to cyH and these values were very similar to those observed under normoxic conditions (Figure S2).

Re-oxygenation periods interspersed with hypoxia periods are a distinctive feature of cyH. This intermittent re-oxygenation might trigger the production of ROS and induce oxidative stress by mechanisms involving the mitochondrial respiratory chain since mitochondria are the main source of ROS in cells [49,50]. Therefore, we decided to examine the impact of the cyH protocol on ROS production. Using DCFH_2_-DA, which in presence of oxidant conditions is transformed to highly fluorescent 2′,7′-dichlorofluorescein (DCF) [51], we found that the maximum levels of ROS were generated in the second and third cycles of the hypoxia/re-oxygenation protocol (one way-ANOVA; F_(8, 36)_ = 6.848; *p* < 0.001; Figure 2E). During the fourth cycle of hypoxia/re-oxygenation, levels of ROS were not statistically different to normoxia (N = 100; H4 = 110.78 ± 2.13; R4 = 116.6 ± 5.42). Intriguingly, the maximum increase in ROS production was found during the R2 period, which was even greater than in H2 (H2 = 115.46 ± 1.93; R2 = 135.60 ± 3.33). These data indicate that cyH induces a burst of oxidative stress in BMMCs that reaches its peak during re-oxygenation, particularly in the second period (R2). Next, we evaluated whether intermittent re-oxygenation was a conditioning factor for the exacerbated increase in ROS production during the R2 period or if a chronic period of hypoxia could induce the same response. With the purpose to mimic the two periods of re-oxygenation (R1 and R2) that cells received in the cyH protocol (see Figure 2A), BMMCs were exposed to chH for 2 h and 1 h of re-oxygenation at the end. Our results show that the increase in ROS production triggered by cyH depends on alternate periods of re-oxygenation, since ROS levels were barely increased compared with normoxia when the two re-oxygenation periods were given together after 2 h of chH (One way-ANOVA; F_(2, 12)_ = 57.09; *p* < 0.001; Figure 2F). Taken together, these data indicate that, in BMMCs, cyH induces the upregulation of hypoxia-related genes such as *Hif-1α* and *Vegf* and an increase in ROS levels that is dependent on alternated periods of re-oxygenation.

Additionally, we found that H2 induced an increase in *Tnf-α*, *Ccl-2*, *Il-6*, *Il-4*, and *Tgf-β* mRNAs when compared with the cells maintained in normoxic conditions (Figure S3). Except for *Tnf-α*, all evaluated cytokines returned to basal expression levels in R2. Surprisingly, we found that cells harvested in R2 exhibited an increase in the *Tnf-α* mRNA levels even greater than in H2 (Figure S3).

After the observation that the most significant changes were found in the second cycle of hypoxia/re-oxygenation (specifically in H2) in our experimental protocol, it was decided to use H2 as the conditions to analyze wide transcriptional changes on BMMCs. In the following experiments, cells in H2 cycle are referred to as cells exposed to cyH.

### 3.3. The Transcriptional Signature of MCs Exposed to Cyclic Hypoxia

**Figure 3:**
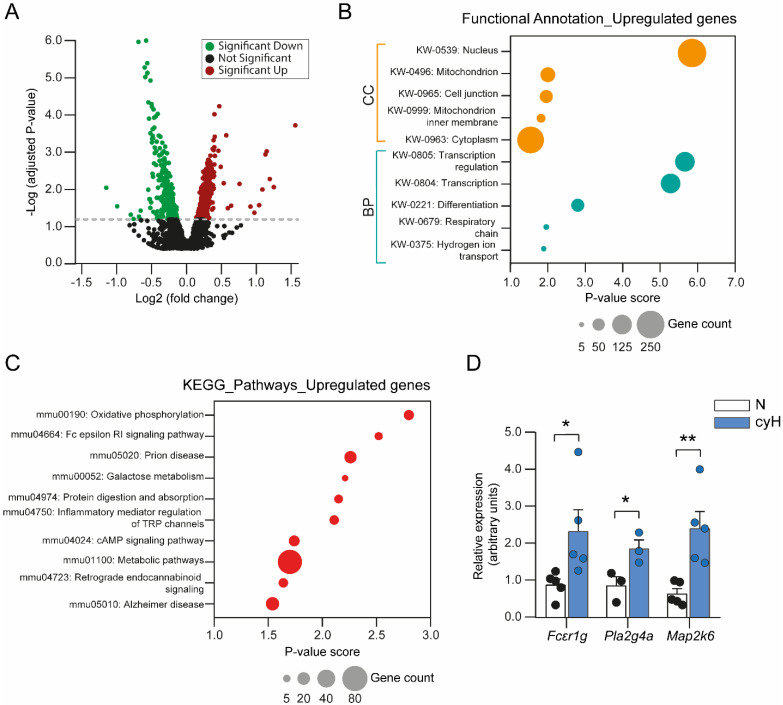
Transcriptional signature of BMMCs exposed to cyclic hypoxia is related to oxidative phosphorylation and the FcεRI signaling pathway. (**A**) Volcano plot of DEGs in BMMCs exposed to cyH. Green dots, downregulated genes; red dots, upregulated genes. (**B**) Functional annotation of upregulated genes in BMMCs subjected to cyH. Enrichment of biological processes (BP) and cellular components (CC) along with the number of genes associated with each functional category. (**C**) Enrichment map of the top ten enriched upregulated pathways in cyH conditions. The number of DEGs associated with each pathway is illustrated according to the size of the circle. (**D**) Two million BMMCs were exposed to N (2.5 h at 21% O_2_) or cyH (H2) and real-time quantitative PCR was performed to validate the overexpression of some DEGs associated with the FcεRI signaling pathway. Unpaired *t*-test, * *p* < 0.05, and ** *p* < 0.01 versus N, *n* = 3–5 experiments for each condition using different BMMCs cultures.

An extensive transcriptomic analysis in MCs subjected to cyH was performed employing microarray technology (see Section 2.10). With that tool, 2512 differentially expressed genes (DEGs), whose Z-score value was greater than 1.5, were found (for details see the Materials and Methods section). The volcano plot shown in Figure 3A displays 1265 upregulated and 1247 downregulated genes by red and green dots, respectively. The upregulated DEGs were clustered and analyzed using the DAVID database, where a total of 950 genes were matched to the mouse genome. Functional analysis with the DAVID database classification system revealed that the transcriptional signature of MCs exposed to cyH is associated with different biological processes (BP), molecular functions (MF), pathways, and cellular components (CC) (Table 1). Particularly, the most enriched BP with the greatest number of genes was the regulation of transcription and the transcription process itself (Figure 3B). Transcripts in this category were from genes involved in DNA methylation, such as *Dnmt1* and *Dnmt3a*; splicing regulation in the hypoxic response such as *Srsf5*, RNA polymerase functions such as *Brf1*, and transcription factors such as members of the *Maf* and the glucocorticoid receptor families *(Nr3c1* and *Nr2e3*) (Table 1). In addition, the most enriched MF was DNA-binding. This suggests that the set of genes upregulated in cyH could be triggering important changes in the transcriptomic profile of MCs that could be associated with epigenetic changes, cell differentiation, and transcriptional network plasticity, as occurs in other immune cells such as macrophages and T cells [52,53,54].

Functional analysis with the KEGG pathway classification system revealed that the most significant enrichment of the MC signature by cyH was the oxidative phosphorylation pathway (Figure 3C). This pathway included many transcripts that encode elements of the mitochondrial electron transport chain and subunits of the ATP synthase, such as *Atp5g2*, *Atp5j2*, and *Atp5l* (Table 1), which would suggest that a compensating mechanism drives MCs to respond to cyH by upregulating genes involved in ATP synthesis. Furthermore, an enrichment of other functional categories such as galactose metabolism, cAMP signaling pathway, reactive oxygen species, and inflammatory response was also found (Table 1). Regarding the cellular localization where most enriched processes were found, most of the overexpressed genes were associated with the nucleus, mitochondria, and cytoplasm (Figure 3B).

Interestingly, the cyH-related MC signature also showed enrichment in the FcεRI signaling pathway (Figure 3C). This category includes transcripts encoding proteins that participate in different steps of the FcεRI signaling cascade (Table 1). For example, *Fcer1g*, which encodes the γ chains of the FcεRI complex and possesses ITAMs that transduce activation signals after cross-linking of FcεRI; *Pla2g4a*, which encodes a member of the cytosolic phospholipase A2 involved in the synthesis of lipid mediators; and *Map2k6*, which encodes a mitogen-activated protein kinase (MAPK) that regulates the activation of several transcription factors leading to *de novo* synthesis of many chemokines and cytokines.

### 3.4. Cyclic Hypoxia Induces an Increase in the Transcription of Genes Encoding Elements of the FcεRI Signaling Pathway, Causing Enhanced Responsiveness to IgE/Ag Challenge

**Figure 4:**
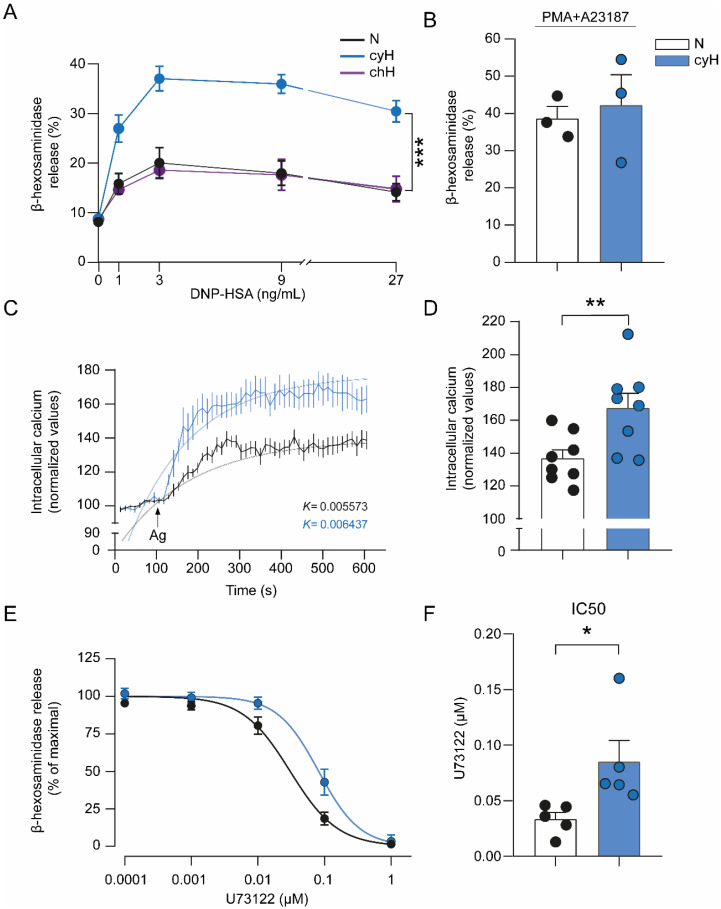
Cyclic hypoxia increases IgE/Ag-dependent degranulation and intracellular Ca^2+^ mobilization, together with an overactivation of PLC-γ in BMMCs. Two million BMMCs were exposed to different conditions and sensitized with 100 ng/mL IgE 1 h before the end of each protocol. Cells were then stimulated with a specific Ag DNP-HSA. (**A**) Release of β-hexosaminidase in BMMCs incubated under normoxic conditions (2.5 h at 21% O_2_, N), cyH (H2), or chH (2.5 h at 1% O_2_) and stimulated with different concentrations of Ag for 30 min. (**B**) Two million BMMCs were exposed to N or cyH and then stimulated with 1 μM of both PMA and A23187 for 30 min. The release of β-hexosaminidase was then determined in the cell´s supernatants. (**C**) Ten million BMMCs were incubated in N or cyH and loaded with Fura 2-AM as described in the Materials and Methods section. Basal Fura 2-AM fluorescence was recorded for 100 s, and then cells were stimulated with 27 ng/mL Ag. Fluorescence was recorded during 600 s and the rate constant, K, was calculated by fitting a monoexponential function. (**D**) Bar graph illustrating the maximum intracellular Ca^2+^ rise under N and cyH conditions. (**E**) Two million BMMCs were exposed to N or cyH and then preincubated with different concentrations of the PLC inhibitor U73122 for 15 min in normoxic conditions. Cells were then stimulated with Ag (9 ng/mL) for 30 min. The percentage release of β-hexosaminidase was measured as described in the Materials and Methods section. (**F**) Bar graph representing the half-maximal inhibitory concentration (IC50) values at which degranulation is inhibited in normoxic and hypoxic BMMCs. Unpaired *t*-test and Two-Way ANOVA, * *p* < 0.05, ** *p* < 0.01, and *** *p* < 0.001 versus N, *n* = 3–5 experiments for each condition using different BMMCs cultures.

As the FcεRI is a master regulator of MC functions and is also considered essential for their differentiation and maturation [55,56], we decided to evaluate this signaling pathway in MCs exposed to cyH. To validate the obtained results in the microarray, quantitative real-time PCR was employed. As expected, the expression of some FcεRI signaling pathway-related genes such as *Fcεr1g*, *Pla2g4a*, and *Map2k6* was increased at least twofold in hypoxic BMMCs relative to normoxic cells (Figure 3D). Next, we evaluated whether cyH could modulate MC physiological responses and enhance the degranulation extent of MCs. Thus, BMMCs were exposed to normoxia or cyH, and the release of β-hexosaminidase, a well-known degranulation marker, was evaluated after stimulation with IgE/Ag complexes. We found that MC degranulation was exacerbated in cells subjected to cyH compared with those that remained in normoxic conditions (RM-Two Way ANOVA; F_(1, 80)_ = 87.290; *p* < 0.001; Figure 4A, blue line). In contrast, when BMMCs were exposed to chH (2.5 h at 1% O_2_), no changes were observed in the degranulation pattern compared with normoxic conditions (RM Two-Way ANOVA; F_(1, 10)_ = 0.015; *p* = 0.906; Figure 4A, purple line). These results put forward the differential molecular mechanisms triggered by chH and cyH, as demonstrated in endothelial cells [48].

The hyperresponsiveness observed in BMMCs under cyH was dependent on FcεRI-triggering, since when a nonspecific stimulus (the PKC activator PMA and a Ca^2+^ ionophore A23187) was used, no changes in degranulation were observed between cells subjected to cyH and those maintained in normoxic conditions (unpaired *t*-test; t_(4)_ = 0.407; *p* = 0.705; Figure 4B). These data indicate that cyH modifies the MC phenotype inducing modifications in the expression of genes related to the FcεRI signaling pathway.

### 3.5. Cyclic Hypoxia-Induced Hyperresponsive Phenotype of MCs Is Associated with an Increase in Calcium Levels and Overactivation of Phospholipase C

To delineate some of the molecular changes involved in the cyH-induced hyperresponsive phenotype in MCs, we analyzed events that are relevant to FcεRI-dependent degranulation. First, cytosolic Ca^2+^ mobilization induced by IgE/Ag challenge in BMMCs exposed to normoxia or cyH was evaluated using the Ca^2+^ indicator Fura 2-AM. As expected, the addition of Ag to IgE-sensitized BMMCs induced a rapid increase in Ca^2+^ mobilization as soon as 50 s after stimulation of cells subjected to normoxia or the cyH protocol (Figure 4C). However, the raising of intracellular Ca^2+^ after FcεRI crosslinking was higher in BMMCs exposed to cyH compared with cells maintained in normoxic conditions, reaching a higher maximum Ca^2+^ peak in cyH-treated cells (normoxia = 136.80 ± 5.22; cyH = 167.47 ± 8.95; unpaired *t*-test; t_(14)_ = 2.996; *p* = 0.001; Figure 4D). Given the exponential kinetics of Ca^2+^ rise, an exponential function was fitted to experimental data to obtain the value of the rate constant K, which indicates how rapidly the maximum level of Ca^2+^ is reached. In BMMCs subjected to cyH, the K value was lower than that observed in normoxic cells, indicating that Ca^2+^ rise was reached faster (Figure 4C).

To determine whether PLC-γ (an important enzyme responsible for Ca^2+^ rise after FcεRI triggering in MCs) could be more active in MCs exposed to cyH, we evaluated the degranulation extent at different concentrations of the PLC inhibitor U73122. We found that U73122 inhibited the release of β-hexosaminidase in a concentration-dependent manner in both normoxic and cyH-treated cells (Figure 4E). However, cells exposed to cyH showed a higher IC50 than the cells maintained in normoxic conditions (normoxia = 0.03 ± 0.006 μM; cyH = 0.09 ± 0.02 μM; unpaired *t*-test; t_(8)_ = 2.566; *p* = 0.033; Figure 4F), exhibiting less sensitivity to the inhibitor effect of U73122. Altogether, these results suggest that cyH modifies the FcεRI-dependent degranulation rate in MCs by mechanisms that involve an increase in the intracellular Ca^2+^ levels and enhanced activity of PLCγ.

**Figure 5:**
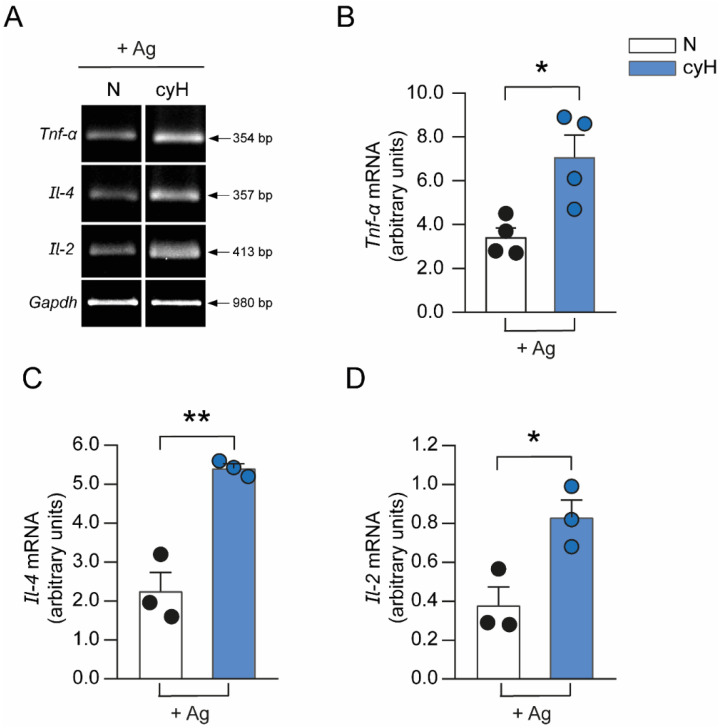
Cyclic hypoxia induces increased mRNA levels of cytokines related to Ca^2+^ signaling in antigen-activated BMMCs. Two million BMMCs were incubated in N (2.5 h at 21% O_2_, N) or cyH (H2) and sensitized with 100 ng/mL IgE 1 h before the end of the cyH protocol. Normoxic or hypoxic IgE-sensitized BMMCs were then stimulated with the specific Ag DNP-HSA (9 ng/mL) for 2 h. Total mRNA was purified to analyze the expression of Ca^2+^ signaling-related cytokines by RT-PCR. (**A**) Representative images of an agarose gel showing amplification of the indicated cytokines. (**B**–**D**) Bar graphs showing densitometric analysis of *Tnf-α*, *Il-4,* and *Il-2* amplicons obtained from normoxic and hypoxic BMMCs after challenge with IgE/Ag. *Gapdh* expression was used as a housekeeping gene. Bar graphs of N and cyH show the fold-change relative to values obtained without Ag stimulation (basal). Unpaired *t*-test, * *p* < 0.05, and ** *p* < 0.01 versus N, *n* = 3–4 experiments for each condition using different BMMCs cultures.

Downstream Ca^2+^ signaling pathways lead to the activation of transcription factors, such as the nuclear factor of activated T cells (NFAT) and the nuclear factor κB (NF-κB), which induce the expression of several cytokines [57]. Figure 5A shows the mRNA expression of Ca^2+^ signaling-related cytokines in BMMCs exposed to normoxia or cyH after the IgE/Ag challenge. As can be observed, the expression of *Tnf-α*, *Il-4*, and *Il-2* was at least twofold in cyH cells compared with normoxic cells (Figure 5B–D). Interestingly, cyH cells did not show changes in the mRNA levels of other cytokines whose expression has been reported to be independent of Ca^2+^ rise (such as *Il-6*, *Ccl-2*, *Il-3*, and *Tgf-β*) when compared with cells maintained under normoxia conditions (Figure S4).

## 4. Discussion

Although the presence of MCs on malignant tumor biopsies has been largely described, their influence on tumor growth remains as an unanswered question, since it ranges from clearly positive to clearly negative depending on the type and the stage of the tumor and the organism where it is studied [58]. Possible explanations for this phenomenon include the potential influence of specific TMEs on MC phenotype, which could lead MCs to synthesize particular mediators able to favor or limit tumor cell replication, blood vessel formation, or intratumoral immune response. To date, the mediators and conditions modulating MC activation in particular TMEs have not been fully described, and potential phenotypic changes on tumor-associated MCs (TAMCs) are largely unknown [13,21,22], although TAMCs seem to constitute a particular subpopulation of MCs that are different to the classical categories that have been used to date [59]. From all the factors present in the TME that activate MCs and can modify their phenotype (such as stem cell factor, adenosine, and IL-33, among others), cyH is one of the least studied.

In the present work, we tested the hypothesis that cyH could alter the transcriptional profile of MCs. To perform this, we first evaluated the presence of MCs in zones subjected to cyH in murine melanoma tumors. Then, an experimental protocol to induce cyH in vitro using BMMCs was standardized in order to, finally, perform an extensive analysis of the transcriptome of cyH-subjected BMMCs. This cell preparation was chosen because several studies have shown that BMMCs express cytokines and proangiogenic factors induced by molecules and conditions present in the TME, and those findings have been replicated in murine tumor biopsies [27,28,30,60]. The expression of some upregulated genes was validated, and the functional consequences of their increased synthesis were evaluated. The main results are the following: (1) MCs are located in tumoral zones subjected to chH and cyH; (2) when tested in vitro, cyH induces transcriptional changes on BMMCs, causing the upregulation of 1265 genes, including those encoding for proteins involved in gene transcription, oxidative phosphorylation, and the FcεRI signaling pathway; (3) cyH triggers functional plasticity in MCs, resulting in a hyperresponsive phenotype of those cells upon IgE/Ag challenge.

TME is a complex interaction niche where genetically transformed and phenotypically adapted cells coexist in hostile conditions. The dynamics of those interactions determine the tumor growth rate, immune escape, and metastatic spreading [61,62]. It has been demonstrated that hypoxia is a characteristic feature of TME and that, in solid tumors, does not occur in a continuous fashion. O_2_ levels fluctuate in a spatiotemporal manner, generating tumor regions with low O_2_ levels surrounded by normoxic regions [26]. Experimental evidence has led to propose that tissue hypoxia can be broadly classified into chronic and cyclic (chH and cyH) [45]. Cyclic hypoxia is a consequence of the highly deregulated intratumoral angiogenesis characterized by the generation of a disorganized and irregularly distributed tumor vasculature that causes periods of intermittent hypoxia associated with leaky blood vessels [24,26,63]. In our study, we observed histological evidence of cyH in murine melanoma tumor biopsies by analyzing the colocalization of hypoxic cells and blood vessel markers (pimonidazole and CD31, respectively). Pimonidazole, a well-established hypoxia marker, is activated by the reduction in its nitro radical specifically in hypoxic cells, forming stable adducts with thiol-containing proteins that can be detected by immunofluorescence [64]. On the other hand, CD31, an adhesion molecule present on the surface of endothelial cells, is considered one of the canonical markers of tumor vasculature [65]. Both markers have been used to detect hypoxic and vascular regions in other murine tumor models [66,67]. In our study, B16-F1 tumors showed immunoreactivity to CD31 in hypoxic zones, an anatomical characteristic of the presence of cyH that has been observed in other tumor types [45].

When the presence and activation of MCs in tumor biopsies were analyzed by tryptase staining, we found that the protease was found completely in cyH areas. Unexpectedly, some tryptase-positive cells did not colocalize with blood vessels, the usual anatomical localization of MCs in normal tissues, suggesting that MCs do not follow the same distribution pattern in normal and tumoral structures. In previous studies, we found the presence of MCs in hypoxic tumor regions and provided evidence indicating those MCs were hypoxic [28]. In the present study, we refined that observation and demonstrate that intratumoral MCs are located in areas subjected to cyH, which could promote particular alterations in their phenotype. Although increased MC infiltration has been demonstrated in many types of solid tumors, including murine melanoma (reviewed in [14]), this is the first study to provide experimental evidence indicating that MCs are exposed to cyH within the melanoma tumor.

To characterize the potential alterations in MC phenotype that could be induced by cyH, an in vitro model to simulate this condition using BMMCs was standardized. Our protocol design was based on previous reports in which in vivo measurements of O_2_ fluctuations in tumor vascular networks revealed a frequency of 0.5 to 1 cycle of hypoxia per hour [68]. The effects of cycles of O_2_ deprivation and re-oxygenation were evaluated on distinct parameters of BMMC physiology, and we found that BMMCs exposed to cyH display an increased expression of typical markers of hypoxia, such as *Hif-1α* and *Vegf*, along with increased ROS production, which is characteristic of re-oxygenation periods. These results are in line with previous studies performed on other cell types such as endothelial cells and tumor cells [47,48], suggesting a conservative function of the HIF-1α-dependent pathway in BMMCs in response to cyH. Interestingly, we found a higher increase in *Tnf-α* mRNA expression in the second re-oxygenation period with respect to the hypoxia period, which could be explained by the greater increased ROS levels in the re-oxygenation periods, since signaling pathways triggered by ROS can activate NF-κB and stimulate the production of proinflammatory cytokines such as TNF-α in other cells [69].

In order to identify transcriptomic changes in BMMCs subjected to cyH, we made use of microarrays technology. KEGG pathway analyses of DEGs in cells subjected to cyH revealed that the upregulated genes were enriched in the oxidative phosphorylation metabolic pathway, including transcripts encoding subunits of the mitochondrial ATP synthase. Furthermore, we found an increased expression in several genes that encode subunits of the mitochondrial electron transport chain, such as the NADH dehydrogenase (complex I), indicating changes in the proteins that contribute to the electrochemical proton gradient required for ATP synthesis. Our findings are consistent with significant energetic changes to deal with the absence of the final electron acceptor for the mitochondrial electron transport chain, as occurs in other cell types [70,71]. The functional implications of this important change in metabolic pathways in MCs are complex and await further investigation.

Analysis of transcriptomic data revealed that the cyH-related MC gene signature was associated with the enrichment of processes such as transcription, transcriptional regulation, and DNA binding. Some of the upregulated genes encode enzymes important for DNA methylation, such as *Dnmt1* and *Dnmt3a*; *Brf1*, *Taf3*, and *Tead1*, which encode proteins required for the transcription initiation complex; or *Srsf5* and *Ang2*, which encode serine and arginine-rich splicing factor 5 and angiopoietin growth factor 2, respectively. Previous studies have reported similar findings in endothelial cells, where increased expression of angiopoietin-2 was found under hypoxic conditions [72]. Interestingly, hypoxia is a condition that alters many alternative splicing patterns of genes, and this process is mediated by several polypeptides, including SR proteins such as SRSF5 [73]. In prostate cancer cells, it was demonstrated that the expression and phosphorylation levels of SRSFs proteins (particularly on SRSF5) are significantly increased in hypoxia [74]. Altogether, our findings contribute to uncovering the ability of MCs to functionally adapt their transcriptional profile to a changing environment, as occurs with other immune cells [52]. To the best of our knowledge, this is the first report evaluating the transcriptional signature of MCs exposed to cyH.

One of the most interesting findings from our study was the enrichment of the FcεRI signaling pathway in MCs exposed to cyH. Eight upregulated genes encoding different proteins of the canonical FcεRI signaling cascade were detected. Since the activation of the FcεRI receptor leads to degranulation and release of preformed and de novo-synthesized inflammatory mediators in MCs that might exert pleiotropic functions in the TME [75], we decided to explore its associated signaling pathway validating, first, three DEGs by quantitative PCR. We found that expression of *Fcεr1g*, *Pla2g4a,* and *Map2k6* was increased in MCs subjected to cyH. Notably, these results were similar to those obtained after the gene expression analysis of synovial membranes from patients with early- and late-stage osteoarthritis and compared with healthy donors. In that study, the expression of genes related to the FcεRI signaling pathway was also upregulated in tissue from osteoarthritic patients. Similarly to our study, an increase on FcεRI gamma and alpha subunits, together with the arachidonate 5-lipoxygenase activating protein, (Alox5ap) was observed [76]. Since osteoarthritis is characterized by tissue inflammation and poor O_2_ perfusion [77], it is possible to speculate that an increase in the signaling elements associated with the FcεRI complex (and, for extension, elevated MC activation) could be a hallmark of low oxygen-bearing environments.

Interestingly, cyH-induced changes go beyond transcriptomic modifications and are translated into hyperresponsiveness to IgE/Ag challenge through the FcεRI/PLC-γ/Ca^2+^ signaling axis. This culminates in an increased release of β-hexosaminidase and induction of immunomodulatory cytokines expression such as *Il-4*, *Il-2*, and *Tnf-α* upon antigenic stimulation. Whether this phenotypic change in MCs subjected to a cyH condition within the tumor may have an impact on tumor development remains an open question that requires further investigation. Several studies suggest that MCs might be hyper-reactive to stimuli present in the tissue microenvironment under pathological states. For example, increased MCs degranulation and tryptase release have been observed in tissue biopsies from osteoarthritis patients, suggesting that the inflammatory and hypoxic microenvironment characteristic of this pathology might activate MCs [76]. On the other hand, it has been shown that LPS activation of murine melanoma-associated MCs induces CXCL10 secretion and promotes T cell recruitment, which, in turn, could enhance immunosurveillance against melanoma tumors [78]. In addition, a recent study demonstrated that MCs exposed to chH (24 h at 1% O_2_) increased the secretion of the chemokine CCL-2, and this secretion was dependent on ROS formation and the translocation of L-type Ca^2+^ channels to the plasma membrane [28]. Interestingly, hypoxic MCs also promoted the translocation of lysosome-associated membrane protein 2 (LAMP2)-positive vesicles from intracellular pools to the plasma membrane [13]. LAMP translocation has been associated with the activation of MCs and basophils [79], since it can be related to the mobilization of granules to the plasma membrane and, eventually, to an increase in MCs degranulation [80].

**Figure 6:**
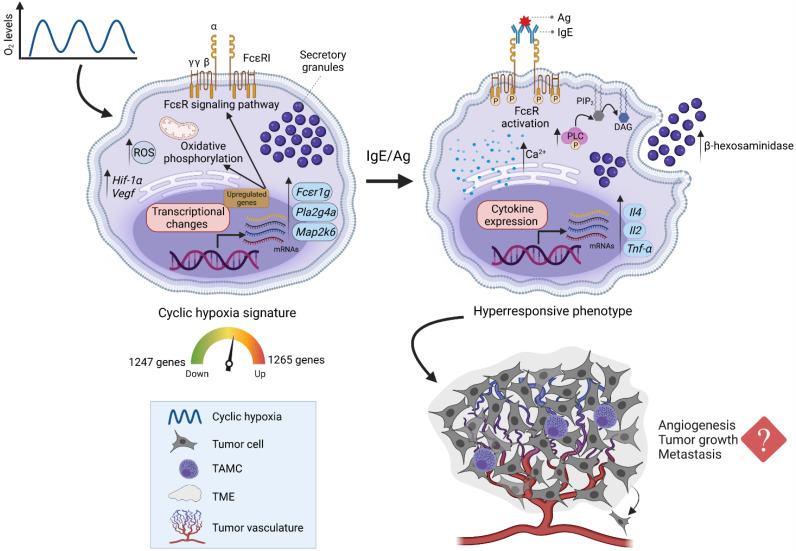
Cyclic hypoxia promotes phenotypic plasticity in MCs due to significant transcriptomic changes that lead to hyper-responsiveness to IgE/Ag-dependent stimulation. MCs exposed to cyH suffer an increase in ROS production and modify their transcriptional profile. Upregulated genes include classical markers of hypoxia such as *Hif-1α* and *Vegf*. Moreover, upregulated genes are associated with oxidative phosphorylation and the FcεRI signaling pathway. An increase in FcεRI-signaling elements leads to increased Ca^2+^ mobilization and overactivation of PLCγ, culminating in enhanced degranulation and expression of cytokine mRNAs, such as *Tnf-α*, *Il-2,* and *Il-4*. This hyperresponsive phenotype of IgE/Ag-activated MCs could promote angiogenesis, tumor growth, and metastasis. TAMC: tumor-associated mast cell; TME: tumor microenvironment. Image was created with BioRender.com (2022).

Evidence suggesting an increase in the activity of the FcεRI signaling system in TAMCs has been found [27]. For example, the increased B16-F1 melanoma tumor growth observed in atopic mice suggests that MCs present in the tumor are more sensitive to IgE-dependent activation, which, in turn, promotes tumor angiogenesis [27]. In addition, a recent study demonstrated that allergies increase tumor growth and resistance to immunotherapy in a histamine-dependent manner [81]. Whether TME-associated MCs might release histamine in response to cyH for promoting tumor growth remains unexplored. Although in this study we showed that MCs exposed to cyH are hyper-responsive after IgE/Ag challenge, results cannot be directly extrapolated to in vivo models of tumor growth, since BMMCs have limitations as a model of fully differentiated cells [82]. Additional studies, for example, isolating TAMCs with the minimal manipulation or single-cell RNA sequencing, are required to know whether the phenotypic change induced by cyH is specific for FcεRI activation or whether it can be extended to other stimuli present in the TME. The main findings of the present work and probable pathophysiological implications are summarized in Figure 6.

## Figures and Tables

**Table 1:** Functional enrichment analysis of cyclic hypoxia-related mast cell signature genes.

Functional Annotation Term	Genes			*p*-Value
Transcription regulation (KW-0805)	*Dnmt1* *Dnmt3a*	*Nr3c1* *Nr2e3*	*Srf5*	$9.2\times 10^{-7}$
Transcription (KW-0804)	*Brf1* *Maf1*	*Maf* *Mafb*	*Ccar1* *Taf3*	$2.2\times 10^{-6}$
DNA binding (KW-0238)	*Aebp2* *Ash2l* *Pou3f3*	*Pou4f1* *Tead1*	*Ang2* *Sox4*	$6.8\times 10^{-5}$
Oxidative phosphorylation (mmu00190)	*Atp5g2* *Atp5j2* *Atp6v1e1* *Atp5l* *Atp4b*	*Ndufs4* *Ndufb5* *Ndufb2* *Ndufb4*	*Ndufa3* *Ndufa5* *Ndufc1* *Cox10*	$1.6\times 10^{-3}$
Galactose metabolism (mmu00052)	*B4galt1* *B4galt2*	*Akr1b7* *Galt*	*Gale*	$6.2\times 10^{-3}$
cAMP signaling pathway (mmu04024)	*Atp1a2* *Adcy4*	*Edn2*	*Ppp1cb*	$1.8\times 10^{-2}$
Chemical carcinogenesis—Reactive oxygen species (mmu05208)	*Cyp1b1* *Gstm6*	*Pik3cb*	*Sod1*	$3.7\times 10^{-2}$
Inflammatory response (KW-0395)	*Bcl6* *Ccl21a*	*Cxcl13*	*Cx3cl1*	$9.5\times 10^{-1}$
Fc epsilon RI signaling pathway (mmu04664)	*Fcer1g* *Alox5ap* *Mapk8*	*Map2k6* *Pik3cb* *Pla2g4a*	*Raf1* *Vav3*	$3.0\times 10^{-3}$

Identifiers in parentheses (left column) were designated by the DAVID functional annotation tool. *p*-values, Fisher’s exact test. The smaller, the more enrichment.

## Data Availability

The data presented in this study are openly available in Gene Expression Omnibus under the accession number GSE201828.

## Supplementary Materials

The following supporting information can be downloaded at: https://www.mdpi.com/article/10.3390/cells11142239/s1, Figure S1: Negative control of immunofluorescence assays in murine B16-F1 melanoma tumors. Figure S2: Cyclic hypoxia does not modify BMMC viability; Figure S3. Cyclic hypoxia modifies the cytokine transcriptional profile in BMMCs. Figure S4: Cyclic hypoxia does not induce increased mRNA levels of *Il-6*, *Ccl-2*, *Il-3,* and *Tgf-β* in Ag-activated BMMCs; Table S1: List of primer sequences used for semiquantitative PCR analysis. References in supplementary materials: [83,84,85,86,87,88,89].
