# Supplementary material for: Cyclic Hypoxia Induces Transcriptomic Changes in Mast Cells Leading to a Hyperresponsive Phenotype after FcεRI Cross-Linking
Source: Cells. 2022 Jul 19;11(14):2239. doi: 10.3390/cells11142239 (PMC9319477; doi:10.3390/cells11142239)
Supplement: Supplementary file 1 [file cells-11-02239-s001.zip › cells-1765367-supplementary.pdf]

Suplemmentary materials

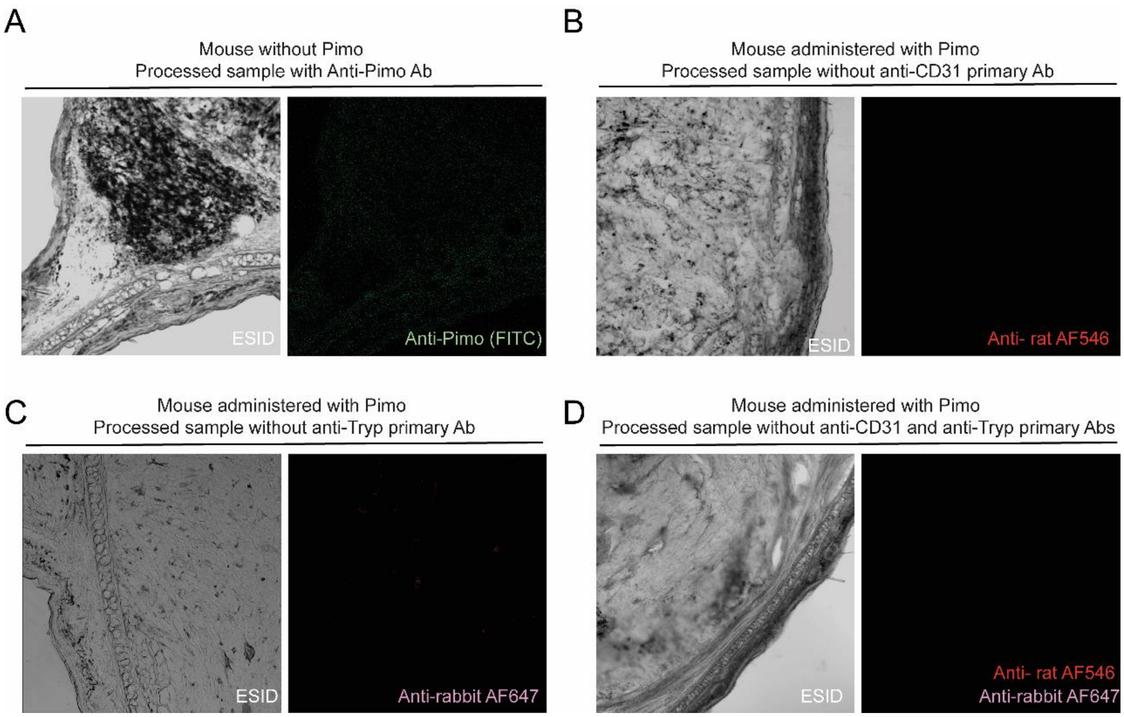

Figure S1. Negative control of immunofluorescence assays in murine B16-F1 melanoma tumor samples.

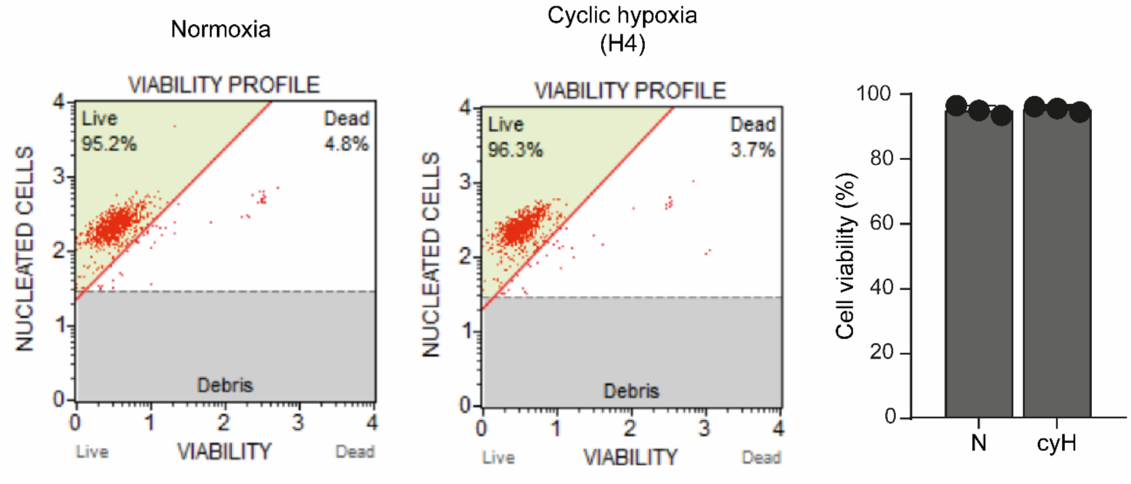

Figure S2. Cyclic hypoxia does not modify MC viability. Two million BMMCs were subjected to N (6 h at 21%O_2) or cyH (four cycles of 1 h at 1% O_2 followed by 30 min re-oxygenation at 21% O_2). Viability of normoxic or hypoxic BMMCs was then assessed as described in the Materials and Methods section. Representative plots indicating the live or dead cell population considering nucleated cells (left panel) together with the bar graph of the percentage of live cells for each condition (right panel). Unpaired t-test, n= 3 experiments for each condition using different BMMCs cultures.

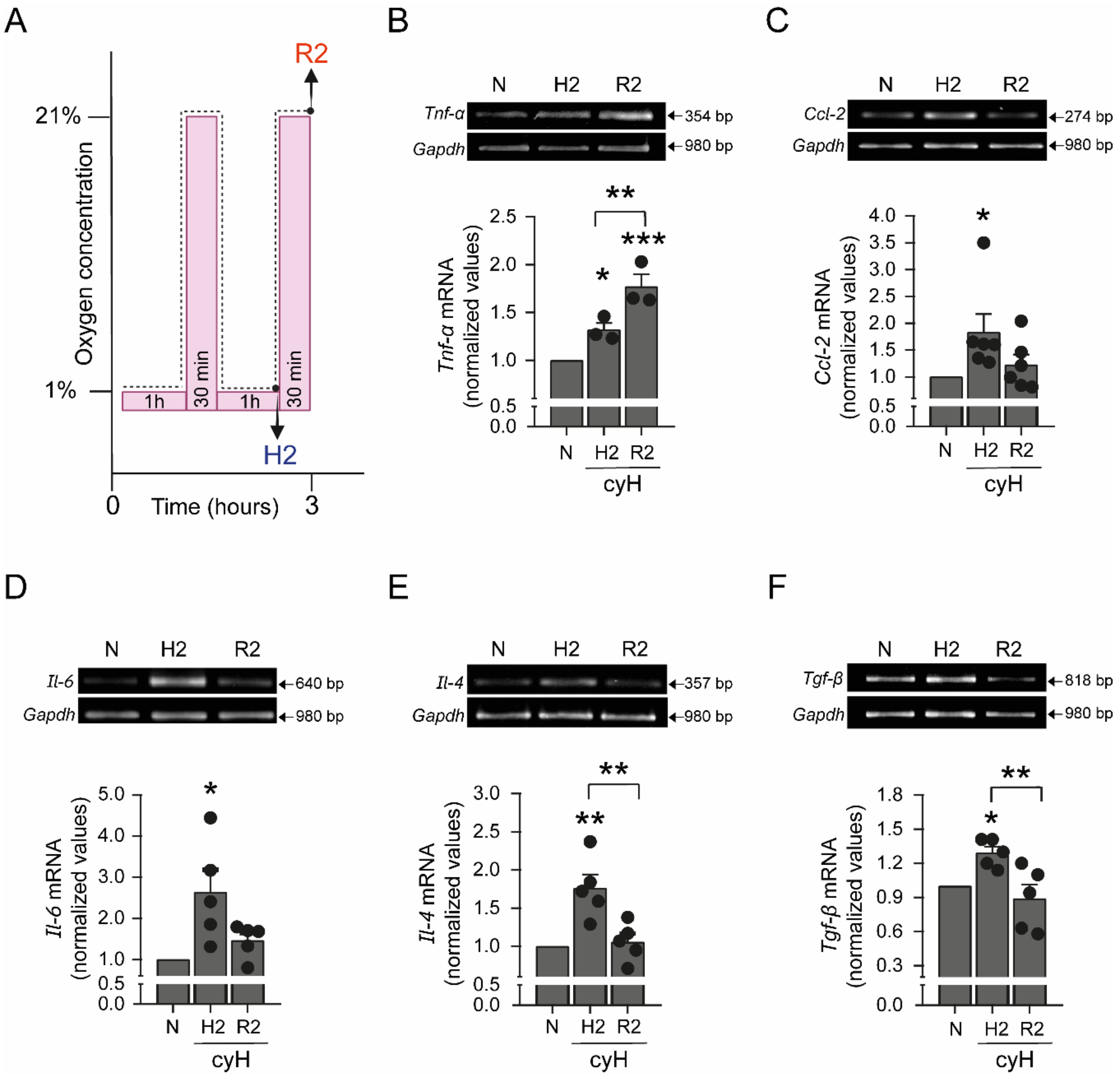

Figure S3. Cyclic hypoxia modifies the cytokine transcriptional profile in BMMCs. Two million BMMCs were incubated in normoxic conditions (3 hours at 21%O_2, N) or cyH (H2 and R2), and total mRNA was purified to analyze cytokine expression by RT-PCR. **(A)** Experimental protocol of cyH illustrating the point at which BMMCs were harvested. **(B-F)** Representative images of agarose gels showing amplification of the indicated cytokine mRNAs, and their densitometric quantification normalized to *Gapdh* expression. One way-ANOVA, *P<0.05, **P<0.01, ***P<0.001 versus N and specified conditions, n= 3-6 experiments for each condition using different BMMCs cultures.

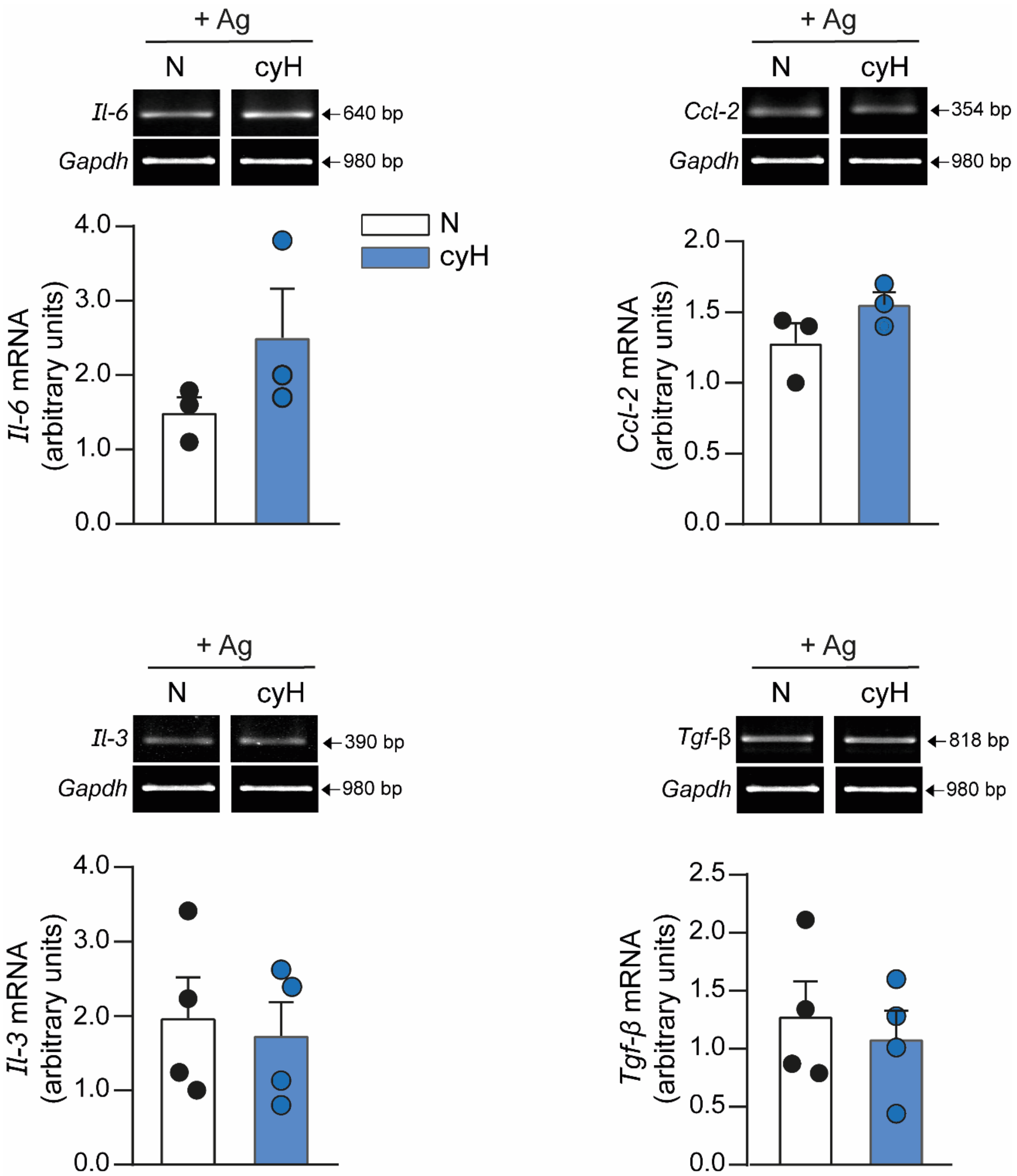

Figure S4. Cyclic hypoxia does not induce increased mRNA levels of *Il-6*, *Ccl-2*, *Il-3*, and *Tgf-β* in Ag-activated BMMCs. Two million BMMCs were incubated in N (2.5 hours at 21% O_2, N) or cyH (H2) and sensitized with 100 ng/mL IgE 1 hour before the end of the cyH protocol. After that, normoxic or hypoxic IgE-sensitized BMMCs were stimulated with 9 ng/mL of Ag (DNP-HSA) for 2 hours. Total mRNA was purified to analyze the expression of the indicated cytokines by RT-PCR. Representative agarose gel images of the amplification of each cytokine and its densitometric analysis are shown. Gapdh expression was used as a housekeeping gene. Bar graphs of N and cyH show the fold-change relative to values obtained without Ag stimulation (basal). Unpaired t-test, n= 3-4 experiments for each condition using different BMMCs cultures.

Table S1. List of primer sequences used for semiquantitative PCR analysis

Gene	Forward sequence	Reverse sequence	Reference
Hif-1α	5′-CTCAAAGTCGGACAGCCTCA-3′	5′-CCCTGCAGTAGGTTTCTGCT-3′	[83]
Vegf	5′-CTGCTCTCTTGGGTCCACTGG-3′	5′-CACCGCCTTGGCTTGTCACAT-3′	[84]
Tnf- α	5′-TTCTGTCTACTGAACTTCGGGGTGA TCGGTCC-3′	5′-GTATGAGATAGCAAATCGGCTGACGG TGTGGG-3′	[85]
Ccl-2	5′-ACTCAAGCCAGCTCTCTCTT-3′	5′-TTCCTTCTTGGGGTCAGCAC-3′	[86]
Il-6	5′-ATGAAGTTCCTCTCTGCAAGAGACT-3′	5′-CACTAGGTTGCCGAGTAGATCTC-3′	[87]
Il-4	5′-CCAGCTAGTTGTCATCCTGCTCTTCTT TCTCG-3′	5′-CAGTGATGTGGACTTGGACTCATTCAT GGTGC-3′	[88]
Tgf-β	5′CGCAACAACGCCATCTATGAGAAA-3′	5′-TTGCAGGAGCGCACAATCATGTTG-3′	*
Il-2	5′-TTCAAGCTCCACTTCAAGCTCTACA GCGGAAG-3′	5′-GACAGAAGGCTATCCATCTCCTCAGA AAGTCC-3′	[88]
Il-3	5′-GATACCCACCGTTTAACCAGAACGTTG-3′	5′-TCCACGGTTAGGAGAGACGGAG-3′	[89]
Gapdh	5′-TGAGGTCGGTGTGAACGGATTTGGC-3′	5′-CATGTAGGCCATGAGGTCCACCAC-3′	[87]

* These sequences were designed in Primer-BLAST (NCBI) in our laboratory.
